# Measuring the Burden: Alcohol’s Evolving Impact on Individuals, Families, and Society

**DOI:** 10.35946/arcr.v35.2.01

**Published:** 2014

**Authors:** Jürgen Rehm, Ralph Hingson

**Affiliations:** **Jürgen Rehm, Ph.D.,***is director of the Social and Epidemiological Research Department at the Centre for Addiction and Mental Health, chair and professor in the Dalla Lana School of Public Health, University of Toronto, Canada, and section head at the Institute for Clinical Psychology and Psychotherapy, Technische Universität Dresden, Dresden, Germany.*; **Ralph Hingson, Sc.D.,***is director of the Division of Epidemiology and Prevention Research at the National Institute on Alcohol Abuse and Alcoholism, National Institutes of Health, Rockville, Maryland.*

Alcohol use is associated with tremendous costs to the drinker, those around him or her, and society as a whole. These costs result from the increased health risks (both physical and mental) associated with alcohol consumption as well as from the social harms caused by alcohol. This issue of *Alcohol Research: Current Reviews* examines the public health impact of alcohol consumption, looking at the full burden of disease that can be attributed to drinking.

The attempt to measure the impact of alcohol use on various disease categories is relatively new to the alcohol research field. In fact, much of our understanding of how alcohol affects health and disease in society is rooted in work from the 1980s and 1990s. This research reflects a truly international perspective. A group of Australian authors, led by Dr. Dallas English, were some of the first to look at the issues involved in attributing mortality and morbidity to substance abuse (English et al. 1995). Dr. James Shultz and his colleagues conducted other seminal research for the Centers for Disease Control and Prevention. They developed the Alcohol-Related Disease Impact (ARDI) software to allow States to calculate mortality, years of potential life lost (YPLL), direct health care costs, indirect morbidity and mortality costs, and non–health sector costs associated with alcohol use (Shultz et al. 1991).Canadian researchers, under the direction of Dr. Eric Single, developed the Canadian version of the alcohol-attributable fractions in the mid-1990s (Single 1999). Also at the forefront of research in this field are Dr. Robin Room and this issue’s Scientific Review Editor, Dr. Jürgen Rehm, both of whom have made significant contributions to our over-all understanding of the field.

The study of the burden of disease on a truly global scale began with the World Health Organization’s (WHO’s) Global Burden of Disease Study (Murray and Lopez 1996). Thanks to the WHO efforts, we now have a worldwide view of the far-reaching consequences of alcohol use and misuse.

Although the field has made much progress and in a short time, there are research gaps that still remain. For example, more research is needed on the relationship between diseases and detailed drinking patterns; more needs to be known about the burden of alcohol-related mental disorders (e.g., depression); future research needs to disentangle the effect of comorbid conditions when assessing the burden of disease attributable to alcohol; the estimates of how alcohol contributes to infectious diseases like HIV need to be further refined; and the alcohol-attributable fractions need to be updated more frequently in response to new developments in science and as the population’s health status and behaviors change. Finally, the impact of alcohol on social harm, including harm to people other than the drinker, still is terra incognita in many areas (Gmel and Rehm 2003).

Over the last two decades, the United States has made substantial progress in improving public health. Still, alcohol remains an important risk factor for disease burden and social harm, not only in the United States but also globally (Murray et al. 2013).Additional research in this area will increase our understanding of alcohol’s role in creating disease burden and social harm and aid in the development of stronger, more effective measures to prevent these devastating effects.
